# Cutaneous metastasis of urothelial carcinoma

**DOI:** 10.1002/iju5.12653

**Published:** 2023-10-19

**Authors:** Alexandra Baczynski, Emily Medhus, Penelope Skopis, Aadil Ahmed

**Affiliations:** ^1^ College of Medicine University of Illinois Chicago Chicago Illinois USA; ^2^ Department of Dermatology Rush University Medical Center Chicago Illinois USA

**Keywords:** cutaneous metastasis, immunohistochemistry, prognosis, skin nodules, urothelial carcinoma

## Abstract

**Introduction:**

Cutaneous metastasis of urothelial carcinoma is a rare occurrence, accounting for a small percentage of skin metastases in cancer patients. This case presentation highlights the importance of considering cutaneous metastasis in patients with a history of urologic malignancy presenting with new dermal nodules.

**Case presentation:**

A 79‐year‐old male with a history of papillary urothelial carcinoma of the bladder and metastasis to the rectum presented with a painful and pruritic rash in the right inguinal region. Physical examination revealed firm papulonodules forming confluent, hyperpigmented to violaceous plaques. A punch biopsy confirmed the diagnosis of cutaneous metastasis of urothelial carcinoma based on histopathological and immunohistochemical findings.

**Conclusion:**

While cutaneous metastasis is uncommon in urothelial carcinoma, early recognition and diagnosis are crucial in guiding patient management and setting realistic expectations regarding prognosis. Timely identification of cutaneous lesions can help facilitate appropriate treatment decisions and discussions of goals of care.

**Abbreviations & Acronyms**
CT: computed tomography
H&E: haemotoxylin and eosin

**Keynote message**

Cutaneous metastasis of urothelial carcinoma, although rare, should be considered in patients with a history of urologic malignancy presenting with new dermal nodules. Timely recognition and diagnosis of these lesions are crucial for appropriate treatment decisions and prognosis discussions, as they can significantly impact patient care and expectations.

## Introduction

The total incidence of skin metastases in cancer patients is estimated to be between 3% and 5%.^1^ Cutaneous involvement of urologic malignancy is lower at only 1.1–2.5%, with bladder cutaneous metastases estimated at 0.84%. The most common site of bladder carcinoma metastasis includes the regional lymph nodes, the liver, lungs, and bone. In patients with a history of urologic malignancy who present with new skin lesions, cutaneous metastasis of underlying carcinoma should be considered.

## Case report

A 79‐year‐old male presented to the dermatology clinic with a new rash. He had a past medical history of chronic kidney disease stage 3, gout, and papillary and invasive urothelial carcinoma, extravesical high‐grade invading into the lamina propria and muscularis propria Stage IVA (pT4b, pN0, cM0) diagnosed by transurethral resection of bladder tumor 05/2020 on pembrolizumab. Of note, his urothelial carcinoma biomarkers were programmed death‐ligand 1 negative (immune cell 5%, tumor <1%), Tempus XF (liquid) = IDH1 GOF, xT = ERBB2, RB1, TP53, CDKN2A, GATA‐3, MTAP, TMB 4.2 m/MB, MS‐S. He was treated with neoadjuvant Gemcitabine/Cisplatin ×3 cycles complicated by sepsis. The patient underwent cystoprostatectomy 09/2020. In 07/2021, he was found to have metastasis to the rectum after sigmoidoscopy biopsies show rectal mucosa and submucosa containing rare foci of malignant cells in lymphatic spaces. GATA‐3 immunostaining was positive in these cells, compatible with a spread from the patient's known urothelial primary carcinoma. Surgery was offered at this time, but the patient declined. He was then started on pembrolizumab. Recent CT and sigmoidoscopy were concerning for the progression of rectal mass. After receiving 15 cycles of pembrolizumab, the patient was started on enfortumab vedotin plus pembrolizumab. On cycle 3 day 1, he presented to dermatology with a rash of the right inguinal region for 5 weeks which was painful and pruritic. He had previously been treated with oral valacyclovir and topical clotrimazole cream with no improvement. The patient denied a history of fevers or night sweats.

**Fig. 1:**
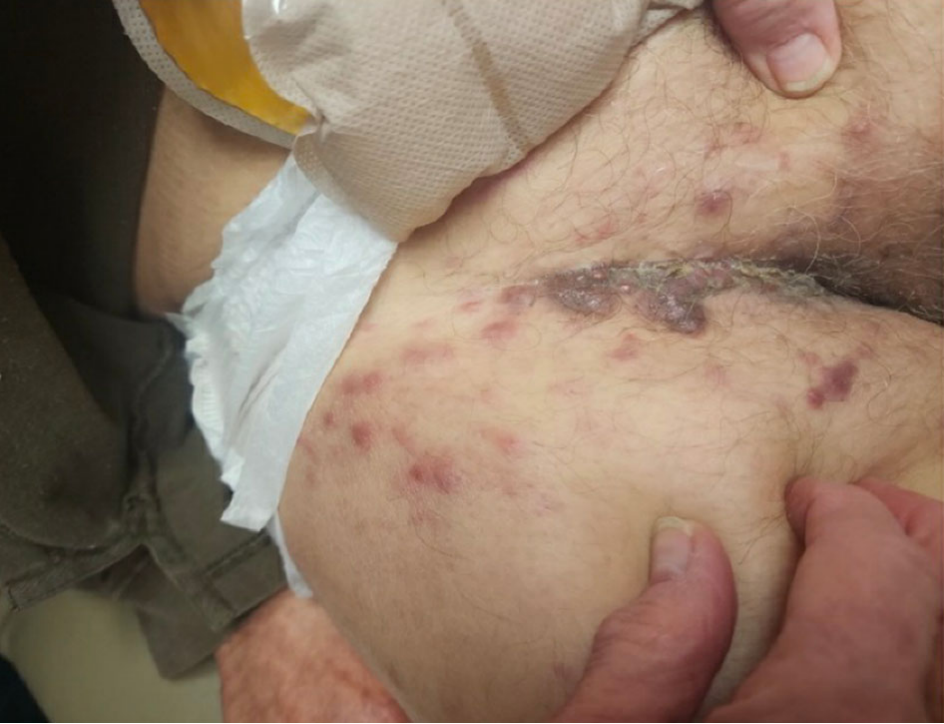
Right inguinal fold with firm papulonodules, several of which form confluent, hyperpigmented to violaceous plaques.

**Fig. 2:**
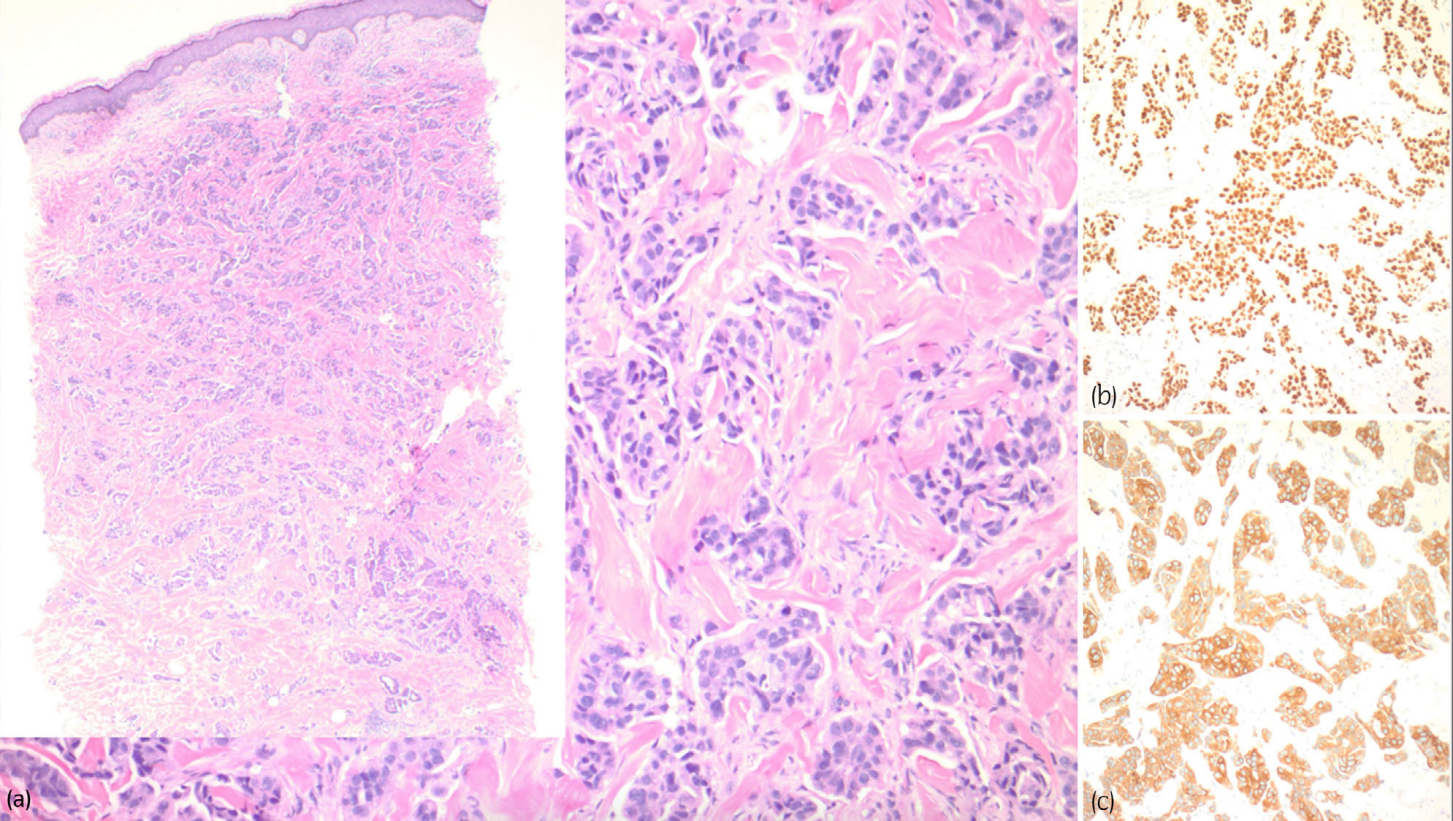
(a) A nested proliferation of tumor cells in the dermis with focal glandular differentiation (H&E, original magnification 100×). The inset image shows skin with papillary dermal edema, perivascular inflammation, and a neoplastic proliferation extending into the deep dermis (H&E, original magnification, 20×). (b) GATA3 stain highlighting the tumor cells with nuclear staining. (Immunostain, original magnification 100×). (c) Cytokeratin 20 stain highlighting the tumor cells with cytoplasmic staining (Immunostain, original magnification 100×).

His physical exam was notable for firm papulonodules, several of which formed confluent, hyperpigmented to violaceous plaques involving the right inguinal fold (Fig. 1). Similar lesions also extended onto the proximal anterior thigh. A punch biopsy was performed which revealed a dermal proliferation of neoplastic cells in irregular nests with retraction artifact and focal glandular differentiation. The cells show high‐grade nuclear features with background apoptosis. There was no involvement of the overlying epidermis. Immunohistochemistry revealed that the tumor cells were positive for CK20 and GATA3 and negative for CK7 (Fig. 2). The presence of a glandular proliferation in the dermis, without overlying connection to the epidermis, and with a staining pattern consistent with the patient's known urothelial carcinoma, led to the diagnosis of cutaneous metastasis. Of note, the papillary dermis showed edema and perivascular inflammation that corroborated the clinical appearance of the rash and can be seen in metastatic lesions, adding to diagnostic challenge on clinical grounds. After confirmation of skin metastasis, the patient received one fraction of intensity‐modulated radiation therapy to the right pelvis/groin. He was continued on enfortumanb vedotin plus pembrolizumab at that time. Significant decline in CA19‐9 and ctDNA had stabilized. The newest CT imaging demonstrates a decrease in abdominal/pelvic lymphadenopathy with no new findings.

## Discussion

The most common cancers associated with metastasis to the skin include 30% of breast cancers, 20% of nasal sinus cancers, 10–20% of melanomas, and 12–16% of cancers in the larynx or of the oral cavity.^2^, ^3^ The proposed mechanism by which bladder cancer is thought to involve the skin includes direct invasion, hematogenous or lymphatic spread, as well as direct seeding iatrogenically.^4^ The clinical morphology of cutaneous metastases of urothelial carcinoma can vary significantly; however, they are often described as firm, nonpainful nodules or infiltrated plaques with nodularity.^5^ In order to definitively diagnose this carcinoma, immunohistochemical studies should be performed. Cytokeratins 7 and 20 are positive in 89% of urothelial bladder carcinomas.^6^ Patients with these metastases to the skin have a very poor prognosis, with a median survival of less than 1 year.^7^ Thus, timely diagnosis of these lesions can help guide goals of care discussions and set expectations for patients and their families.

Even though urothelial carcinoma of the bladder is not typically associated with cutaneous metastases, it is imperative to keep this manifestation on the differential diagnosis because it not only avoids unnecessary treatment – as in this case with valacyclovir and clotrimazole – but also because it has a role in guiding expectations for cancer patients' life expectancy. Although these cutaneous lesions can present in a variety of ways, they are typically nonpainful nodules that can occur throughout the skin. Because urothelial carcinoma can progress as either synchronous or heterochronous patterns, it is likely that the cutaneous metastasis in this patient is a result of the latter.^8^ In addition, recent CT imaging performed during the time of diagnosis of cutaneous metastasis did not identify evidence of other synchronous malignancies within the urinary system.

Histologic differentials include primary cutaneous sweat gland carcinoma and metastasis (urothelial, prostate, breast, salivary glands, etc.). Differentiating cutaneous metastasis from a primary cutaneous sweat gland carcinoma is primarily achieved through clinicopathologic correlation, as histologic findings and immunohistochemistry can overlap. In our patient with known urothelial carcinoma, the presence of a glandular proliferation in the dermis without connection to the overlying epidermis and tumor cells with the same immunophenotype as the underlying malignancy (positive CK20 and GATA‐3), was most suggestive of metastatic disease. The proliferation also lacked features of apocrine glands, as no decapitation secretion was observed, making a primary apocrine carcinoma unlikely.

Due to the dermatomal distribution of our patient's clinical findings, he was first treated for herpes zoster, delaying the ultimate diagnosis. Interestingly, there are many cases of zosteriform or dermatomal eruptions of cutaneous metastasis.^9^, ^10^ There are a total of seven cases of zosteriform cutaneous metastasis of urothelial carcinoma in the literature to date.^10^ Our case is helpful in describing the rare presentation of urothelial carcinoma presenting in a dermatomal distribution. One differentiating factor between a shingles outbreak and zosteriform cutaneous metastasis is the presence of markedly firm nodules and a lack of vesicles, as seen in our patient. When uncertain, a biopsy can be a useful tool to further elucidate the diagnosis.

## Author contributions

Alexandra Baczynski: Writing – original draft. Emily Medhus: Writing – original draft; writing – review and editing. Penelope Skopis: Conceptualization; project administration; writing – original draft. Aadil Ahmed: Data curation; formal analysis; resources; visualization; writing – review and editing.

## Conflict of interest

The authors declare no conflict of interest.

## Approval of the research protocol by an Institutional Reviewer Board

Not applicable.

## Informed consent

Consent was obtained from the patient.

## Registry and the Registration No. of the study/trial

Not applicable.
